# A Novel French-Style Salad Dressing Based on Pickering Emulsion of Oil-Water Lycopene from Guava and Cellulose Nanofibers

**DOI:** 10.3390/molecules29215118

**Published:** 2024-10-30

**Authors:** Catalina Gómez-Hoyos, Angélica Serpa-Guerra, Shaydier Argel. Pérez, Jorge Andrés Velásquez. Cock, Lina Vélez-Acosta, Piedad Gañán-Rojo, Robin Zuluaga-Gallego

**Affiliations:** 1 Programa de Ingeniería en Nanotecnología, Universidad Pontificia Bolivariana, Circular 1—No 70-01, Medellín 050031, Colombia; shaydier.argel@upb.edu.co (S.A.P.); jorgeandres.velasquez@upb.edu.co (J.A.V.C.); 2 Facultad de Ingeniería Agroindustrial, Universidad Pontificia Bolivariana, Circular 1—No 70-01, Medellín 050031, Colombia; angelicamaria.serpa@upb.edu.co (A.S.-G.); lina.velez@upb.edu.co (L.V.-A.); robin.zuluaga@upb.edu.co (R.Z.-G.); 3 Facultad de Ingeniería Química, Universidad Pontificia Bolivariana, Circular 1—No 70-01, Medellín 050031, Colombia

**Keywords:** vinegar, lycopene, nanocellulose, Pickering emulsion, agro-industrial residues

## Abstract

The objective of this research was to assess the potential of a Pickering emulsion based on lycopene extracted from guava by sunflower oil-water and cellulose nanofibers (CNFs) isolated from banana residues as a novel ingredient for a French-style salad dressing. The aim was to determine the impact of this emulsion on the stability and rheological properties of the dressing as well as ascertain the presence of lycopene in the final product. The particle size distribution, rheological properties, and emulsion stability of the Pickering emulsion and salad dressing were evaluated. The sample exhibiting the optimal stability condition contained 0.5 wt.% of CNFs (EPI0.5). In order to prepare the French salad dressing based on this Pickering emulsion, three concentrations of vinegar were analyzed. All samples contained white salt and sugar. The findings suggest that alterations in emulsion stability may be influenced by the vinegar content and the presence of salt, particularly during the storage period, which also affects the concentration of lycopene. Notwithstanding these findings, the untrained panelists expressed a favorable opinion and acceptance of the dressings, indicating that the product could serve as an alternative means of enriching food through the incorporation of beneficial substances such as lycopene.

## 1. Introduction

Despite personal preferences, cultural traditions, and budgetary considerations, the inclusion of foods such as fruits, vegetables, grains, dairy products, proteins, and oils is recommended for a healthier diet [1]. Salads appear as a feasible and culturally sound option to include a wide array of these nourishments in the modern diet [2]. To enhance their flavor and appeal, sauces, such as salad dressings, added to salads, make healthy foods more enjoyable to eat [3] while also altering the flavor of the salad [4].

Salad dressings are classified as oil-in-water (O/W) emulsions [4], typically formulated with ingredients such as egg yolk, vinegar, lipids (fats, oils, or waxes), and spices [4]. Lipids, in particular, play a crucial role in influencing the taste, appearance, texture, and shelf life of the dressings [4]. Salad dressings face several significant challenges including: (1) phase separation of ingredients during preparation or storage, which can negatively impact their shelf life and rheological properties; (2) oxidative degradation through auto-oxidation [5]; and (3) a high fat content, which is associated with an increased risk of health conditions such as diabetes, obesity, and cardiovascular diseases [6]. While reducing the fat content may mitigate these health risks, it can also compromise the sensory attributes and physicochemical stability of the salad dressing [4].

Several strategies have been proposed to overcome these challenges, including the incorporation of proteins and polysaccharides [7]. The success of these interventions depends on their effects on the physicochemical and rheological properties of the dressing as well as their acceptance by consumers [4,7].

In the case of proteins used as fat mimetics, they exhibit textural, rheological, and sensory characteristics similar to those of fat. In contrast, polysaccharides contribute to the formation of a gel-like matrix that affects the creaminess and flow properties [4,8].

Some of the proteins considered for use as fat mimetics include whey protein isolate, which has been extensively analyzed [9]. Polysaccharides such as starch, cellulose derivatives, cellulose, maltodextrins, and pectins have also been evaluated [8]. In certain cases, agro-industrial residues, such as hot pepper seed oil, can provide both proteins and cellulose fibers [4].

On the other hand, a diet rich in carotenoid-containing foods is recommended due to their antioxidant properties [5] and their potential role in the prevention of various types of cancer [10]. However, the bioaccessibility of carotenoids in vegetables is notably low. Research indicates that the intestinal absorption of carotenoids is enhanced by the presence of fat [11]. This supposes the necessity of the inclusion of the amount of lipids into salad considering the effect on improving the release of carotenoids from plant matrices [11] such as, for example, vegetable oil [12].

Given these considerations for incorporating lycopene into salad dressings, several strategies have been explored, including: (1) the addition of vegetables or fruits that provide lycopene and soluble fibers, such as Gac fruit (*Momordica cochinchinensis Spreng*) [5]; (2) the use of biosurfactants that promote the formation of carbohydrate–protein–lipid complexes; and (3) the development of oil-in-water emulsions containing oil-soluble carotenoids [12].

The quantity of lycopene integrated into a dish is dependent upon the food matrix, specifically the fat content. Due to its non-polar nature, it is considerably more straightforward to incorporate substantial quantities of lycopene into fatty meals, such as salad dressing, butter, or chocolate, than in predominantly aqueous foods, including soft drinks, tea, or alcoholic beverages [13].

The application of Pickering emulsions with nanocellulose as a substitute for protecting bioactive compounds, such as lycopene, within salad dressing formulations remains understudied, particularly when the nanocellulose is derived from agro-industrial residues. Furthermore, it can be regarded as an emulsifier for salad dressing [14] and a source of dietary fiber. One example is the utilization of cellulose nanofibers (CNFs) derived from lime residue powder as a stabilizer in Pickering emulsions in salad dressings containing egg yolk [14].

In light of our previous research on the extraction of lycopene from guava using sunflower oil [15] and the use of CNFs from banana rachis as solid particles for stabilizing the Pickering emulsion [16], this study explored the preparation of a French-style salad dressing based on a Pickering emulsion containing lycopene from guava and CNFs from banana rachis. The Pickering emulsion and salad dressing were evaluated through a multi-faceted approach that included photograph registration, light microscopy, and viscosity measurements. Furthermore, a sensory evaluation of the salad dressing was conducted to ascertain consumer acceptability.

## 2. Results and Discussion

### 2.1. Pickering Emulsions

The CNFs used in this work consisted of an entangled network of fibrils and fibril bundles, where most of their diameters were between 5 and 100 nm, with a small amount of subfibrillated material above this range (Supplementary Figure S1). As observed in Supplementary Figure S1, CNFs have a high aspect ratio and form an entangled network which allows the formation of stable emulsions without creaming or sedimentation during storage. CNFs form a strong three-dimensional network that “encapsulates” the oil drops in emulsions [16]. This three-dimensional network forms a gel structure that limits the flow of oil droplets through the water phase [17]. The formation of this network, with a gel-like behavior, was evidenced for CNFs from banana rachis, in a previous work [16].

**Figure 1:**
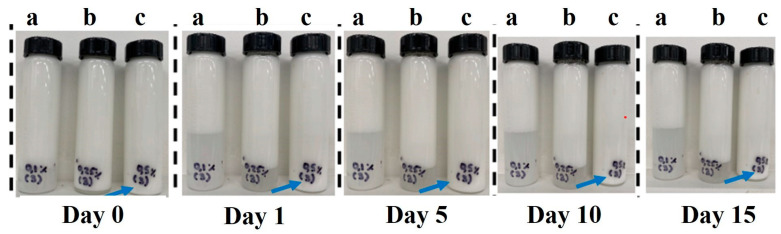
Physical stability of Pickering emulsions at different days of storage by microscopic observation. (**a**) EPI0.1; (**b**) EPI0.25; (**c**) EPI0.5.

To evaluate the effect of the concentration of CNFs on the emulsions, the physical stability was monitored by a photographic registry. Figure 1 depicts the separation of the serum layer from the emulsions, after one day of storage in EPI0.1 and EPI0.25, showing the effect of low concentrations of CNFs indicated by the formation of two layers, while at a high concentration, EPI0.5, the higher amount of CNFs promoted the stabilization of the oil phase, avoiding its phase separation [18].

The two layers that formed in EPI0.1 and EPI0.25 (clear phase at the bottom of the flask and a white suspension at the top) indicate a creaming process [19]. This occurs when droplets flocculate, forming clusters without losing their identity to form larger droplets or coalescing. Since the clusters have a lower density than the water phase, they float to the top of the emulsion [20,21]. From Figure 1, it appears that the increasing content of CNFs increased the stability of the emulsion, as the creamed volume was lower for EPI0.25 than for EPI0.1, possibly due to an increase in the viscosity of the surrounding medium, resulting in slower flocculation or coalescence of the oil droplets [20,21]. Separation of the oil phase for EPI0.5 was not detected during the duration of the study, indicating that no emulsion breakage occurred.

**Figure 2:**
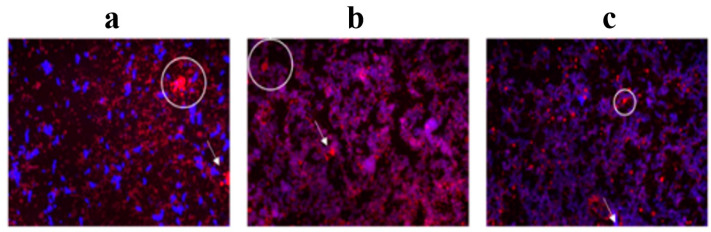
Microstructure of Pickering emulsions observed by fluorescence microscopy. (**a**) EPI0.1; (**b**) EPI0.25; (**c**) EPI0.5.

The microstructure of the emulsion was monitored one day after processing by fluorescence microscopy, in accordance to the methodology described by Bai et al. (2018) [20]. As illustrated in Figure 2, in addition to the three-dimensional self-assembled structures, gels with high viscosity were formed that prevent droplet coalescence. The emulsifying capacity of CNFs was also due to its amphiphilic surface nature and anisotropic behavior, which requires a low solid content for the formation of self-assembled structures [22].

However, the CNF concentration should be adjusted to ensure the coverage of all droplet surfaces and avoid coalescence [16]. It has been reported that different concentrations of CNFs can be used to stabilize O/W emulsions. In a study by Velásquez-Cock et al. (2021) [16], 0.7 wt.% CNFs from banana was found to provide more coverage to coconut oil droplets and restrict their coalescence. The appropriate concentration of CNFs depends on the physicochemical characteristics of the emulsion phases. The formation of coalesced structures is a result of smaller droplets coalescing to form a larger droplet due to their affinity [23]. It is notable that coalesced droplets were more evident in EPI0.1 and EPI0.25 (white arrows in Figure 2a,b). These coalesced droplets coexisted with oil droplets entrapped in the CNFs. The occurrence of coalescence was less prevalent in EPI0.5 (Figure 2c). As illustrated in the stabilization analysis (Figure 1), this sample exhibited minimal droplet destabilization largely due to the entrapment of oil within the CNF network-formed flocs. As observed in the microstructure of the formulated emulsions (Figure 2a–c), a CNF network (blue) did not cover each drop (red). Instead, it appeared to adsorb the neighboring droplets, forming a “droplet network” that was stable when 0.5 wt.% of CNFs was used [16,18,24,25,26].

Considering these results, the Pickering emulsion stabilized with CNFs, with a composition of 0.5 wt% (EPI0.5), was selected as the basis for preparing the French-style salad dressing.

To supplement the analysis of the physical stability and interfacial interaction between the CNFs and droplets observed by physical stability and fluorescence microscopy for the EPI0.5 sample, and considering that understanding the emulsification process requires the measurement of the droplet size distribution of the oil phase as well as knowledge of its viscous and viscoelastic properties, all of which are related to the stability of the emulsion.

**Figure 3:**
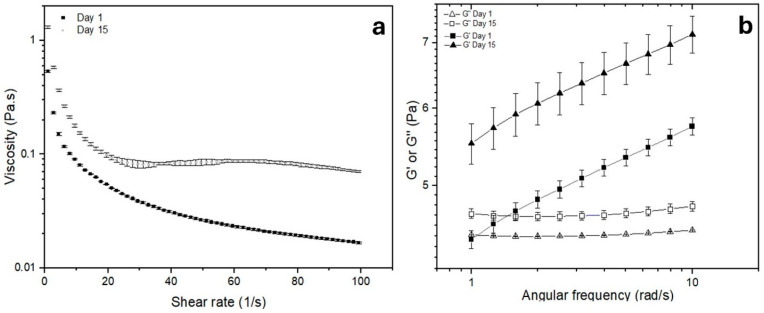
Rheological evaluation of the EPI0.5 sample. (**a**) Viscosity; (**b**) frequency sweep, with G′ represented by filled symbols and G″ by empty symbols.

As shown in Figure 3a, the apparent viscosity decreased with increasing shear rate; this behavior can be associated with a pseudoplastic fluid, possibly due to the breakdown of the three-dimensional entangled network and the fibril orientation along flow lines upon increasing the shear force [27]. Values from the flow curves were fitted to the Ostwald-de Waele model: consistency index, flow behavior, and R^2^ are summarized in Table 1.

As seen in the results of Table 1, the model was adequate to describe the flow behavior of the emulsions (R^2^ values). Additionally, the storage time led to an increase in the consistency index (K), indicating greater firmness of the sample, while the flow index (n) showed the opposite tendency, indicating a more pseudoplastic behavior. This behavior has been reported for other Pickering emulsions [28] and is being related to the process used to obtain the Pickering emulsion, in this particular case, high pressure homogenization.

The results of the viscoelastic properties of the emulsion reported in Figure 3b allowed us to establish that this sample could be classified as a gel-like emulsion, since the storage modulus G′ was greater than the loss modulus G″ at all angular frequencies, thus in the emulsion, elastic properties are dominant rather than viscous ones. The CNFs’ three-dimensional network around droplets after high pressure homogenization might play a prominent role in the observed behavior [28]. Additionally, the observed increase in G′ and G″ after storage corresponds to an enhanced gel strength [29].

As shown in Table 2, the EPI0.5 emulsion presented a strong negative charge and high magnitude of ζ-potential values that were greater than −30 mV. These potentials are associated with stable emulsions during storage, suggesting greater electrostatic repulsion between the droplets and good stability of the emulsion [30]. The same behavior was observed for particle size D [3,2], indicating that in CNF0.5, no droplet coalescence occurred during storage. In summary, the stability reached for CNF0.5 can be attributed to three mechanisms: (i) the high aspect ratio and three-dimensional network of CNFs; (ii) adsorption of the CNFs at the oil droplet surfaces based on Pickering stabilization, and (iii) strong electrostatic repulsion (ζ-potential) [20].

As previously noted, Pickering emulsions have been identified as a viable alternative for the protection of a range of bioactive compounds including curcumin, limonene, eugenol, phytosterols, polyphenols, and carotenoids [31]. As shown in Table 2, the lycopene content in the stabilized emulsion remained consistent throughout the storage period. These findings demonstrate the potential of CNFs as a lycopene protection solution. The interfacial layer formed during Pickering emulsion processing provided efficient lycopene protection, and the compactness of Pickering emulsions inhibits oxygen contact [32], which may explain the observed stability of lycopene. Furthermore, this finding is related to the total color change observed throughout storage, as lycopene was the chemical responsible for the emulsion’s color. For this characteristic to be perceived by an average observer, the color delta (∆E) must be greater than 4, and the results obtained were below this numerical value [33], as reported in Table 2, implying that the emulsion displayed a stable color after the storage period.

Even though there are promising applications of CNFs as Pickering emulsions, their behavior under practical conditions needs to be evaluated, as changes in pH or ionic force could impact the stability of the emulsion.

### 2.2. French-Style Salad Dressing

**Figure 4:**
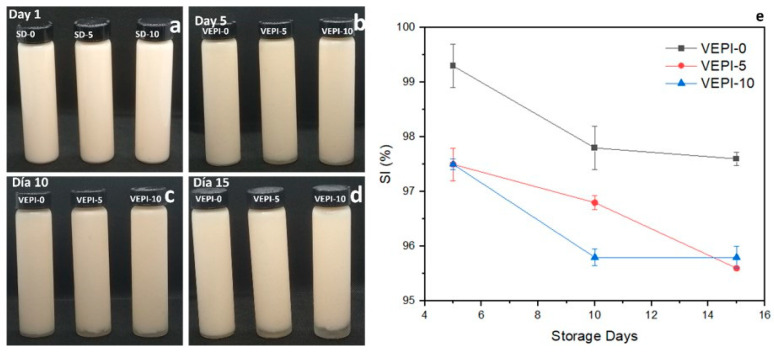
Physical stability of French-style salad dressing at different days of storage by microscopic observation. (**a**) Day 1, (**b**) Day 5, (**c**) Day 10, (**d**) Day 15, (**e**) stability index (SI).

Based on the results and the protective effect of CNFs on lycopene, three French-style salad dressing formulations were prepared using different vinegar proportions. As shown in Figure 4a–d, CNFs assisted in the formation of stable dressings during the initial days of storage; however, photographic follow-up revealed that by the fifth day of storage, all samples exhibited a defined interface (aqueous and oil), and the amount of separation rose with storage duration. In other words, droplets that were uniformly distributed in the emulsified system following mechanical treatment began to concentrate in the upper area of the container as storage time passed, resulting in lower sedimentation rates, as shown in Figure 4a–d.

The change described above and reported in Figure 4e related to the variation in the SI was more noticeable in samples containing vinegar and salt, VEPI-5 and VEPI-10, which had statistically comparable sedimentation rates of less than 96% at the end of storage. In other words, the addition of vinegar to the French-style dressing resulted in a change in its physical stability. It is possible that the reduction in SI for all samples of the French salad dressing may also have been affected by the addition of salt. This destabilization behavior may be attributed to the sensitivity of the emulsions to alterations in ionic strength (salt addition) and pH of the continuous phase, as previously reported by other authors [34,35].

The phase separation observed in Figure 4a–d can be attributed to emulsion destabilization processes, including the creaming phenomenon resulting from the flocculation of oil droplets associated with hydrophobic interactions, van der Waals forces between cellulose nanofibers, and ionic forces associated with pH change and the presence of salts. These observations align with the aforementioned explanation [34,36].

**Figure 5:**
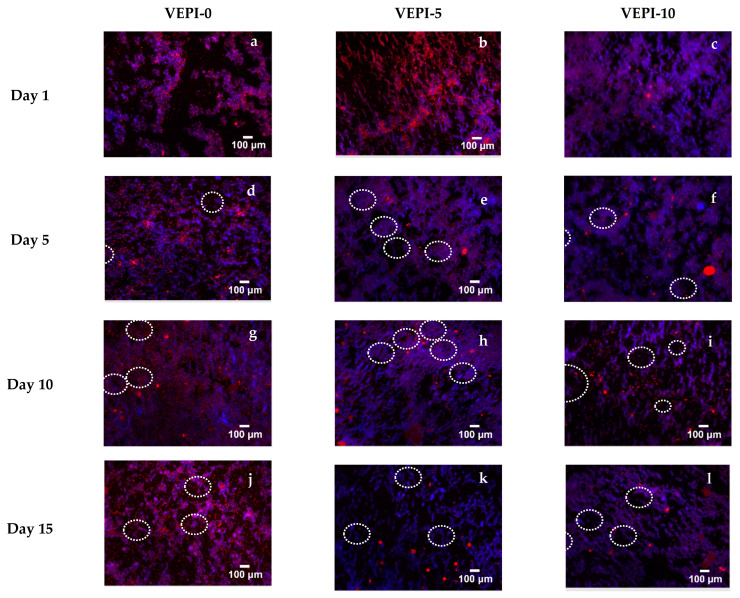
Microstructure of French-style salad dressing observed by fluorescence microscopy.

Figure 5a–l shows the fluorescence microscopy images of the French-style dressing during the storage period. The images presented illustrate the fusion of the blue channel, calcofluor white (cellulose nanofibers), and the red channel, Nile red (sunflower oil droplets enriched with lycopene), and show changes in the microstructure of the emulsion. The results show that the samples consisted of an emulsion in which the oil phase (red) was dispersed in an aqueous phase and remained stable with cellulose nanofibers (blue) at the interface. This property was maintained during storage.

Fluorescence microscopy revealed changes in the microstructure of the emulsion, which can be related to phase separation, as there was an insufficient amount of cellulose nanofibers to stabilize the entire interfacial area of the sunflower oil droplets enriched with lycopene. As observed in the fluorescence images shown in Figure 5 after day 5 of storage, large oil droplets, red areas (circled in white), were observed, corresponding to the creaming of the oil droplets. These droplets were observed in greater proportion for the VEPI-5 and VEPI-10 emulsions, which had an aqueous phase composed of water with salt ions (Na^+^ and Cl^−^) and acetic acid ions (CH_3_CO_2_^−^ + H_3_O^+^). The mixture of these two substances formed sodium acetate with an ionic strength different from that of the salt present in the VEPI-0, which caused greater destabilization of the formulated French-style dressing.

**Figure 6:**
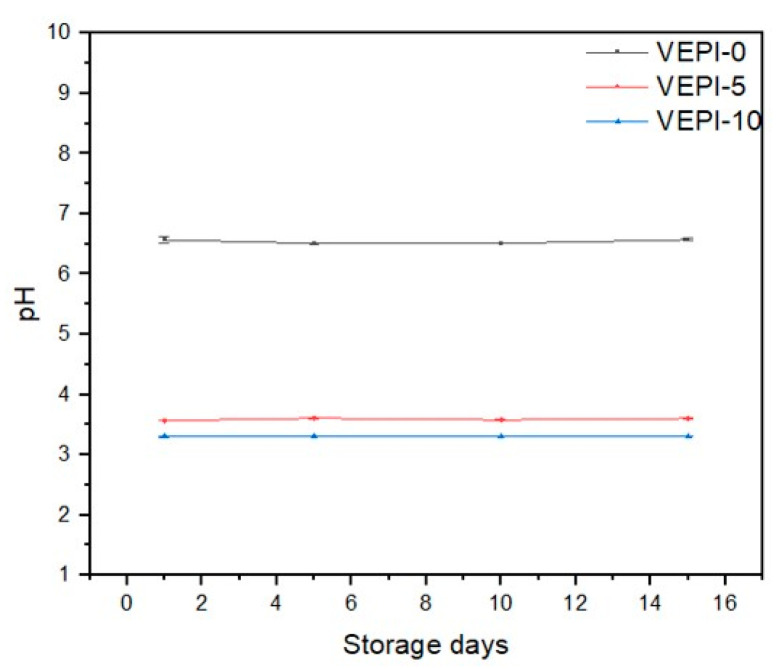
Evaluation of the pH of the French-style salad dressing during storage.

As shown in Figure 6, samples VEPI-5 and VEPI-10 showed a significant decrease in pH compared to sample VEPI-0 due to the presence of fruit vinegar in the aqueous phase of these French-style dressing. White vinegar is acetic acid obtained after acetification of the alcohol obtained after the fermentation of various fruits [37]. The dissociation of acetic acid in water increases the H^+^ concentration, which decreases the pH. As a result, the pH was higher in VEPI-10 than in VEPI-5. In contrast, sample VEPI-0 presented a pH close to neutral due to the fact that this sample was formulated without acetic acid, as indicated in the Section 3. Materials and Methods section.

The emulsions formulated as a vinegar matrix differed from the Pickering emulsions (EPI samples) not only in terms of the change in pH, but also by the presence of salt ions (NaCl) and sucrose molecules present in the French-style dressing. It has been reported that the stability of Pickering emulsions can be affected by changes in pH and ionic strength. The presence of salt ions and acetic acid in the aqueous phase contributes to the destabilization of the emulsion because it favors the destabilization of H^+^ present in the hydroxyl groups of the cellulose nanofibers, which causes a destabilization of the nanofiber network that forms to stabilize the emulsion [38]. In addition, the presence of salt reduces the electrostatic repulsion between oil droplets after the addition of salt due to electrostatic shielding effects [35].

**Figure 7:**
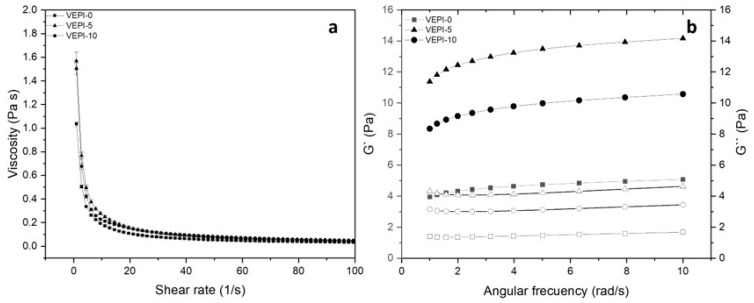
Rheological evaluation of French-style salad dressing. (**a**) Viscosity; (**b**) frequency sweep, with G′ represented by filled symbols and G″ by empty symbols.

To verify that the CNFs acted as an emulsion stabilizer, the rheological behavior of the French-style salad dressing is reported in Figure 7. In terms of the rheological properties, salad dressing is a complex system that shows a time-dependent behavior, as was the case of EPI. The characterization of such behavior in salad dressings is important for food processing and handling, process design and control, product development, structure and flow relationship, physical parameters, and sensory evaluations [39].

All samples showed a decrease in apparent viscosity with an increasing strain rate (Figure 7a). Such behavior is characteristic of non-Newtonian fluids such as pseudoplasticity. In addition, the consistency of a fluid is related to the interactions between the particles that make it up [40], in this case, the interactions between water/vinegar, oil, and cellulose nanofibers. Considering that three samples had the same content of cellulose nanofibers and that the observed results for viscosity did not show substantial changes, it can be concluded that interactions with the nanofibers were mainly responsible for the consistency of the samples. These nanofibers had a larger surface area than cellulose, which contributed to an increase in interfibrillar forces due to van der Waals bonding and intermolecular hydrogen bonding, resulting in a higher resistance to flow (i.e., more viscous samples).

This was confirmed by the results obtained by fitting the curves with the power law (Table 3), where the values of the consistency index (K) and the flow behavior index, n, showed small changes related to the agglomeration generated by pH and the stabilizing effect of the acetate ion over uncharged CNFs [41], resulting in slightly more pronounced pseudoplastic behavior at higher acetic acid contents. These indices allow for differences to be detected between the consistencies of the different samples, since at higher viscosities, large increases in K and reductions in n are expected.

To evaluate the behavior of the formulated French-style dressings, their zeta potentials are listed in Table 4. In general, the measured Z-potential values from the day of emulsion formulation to the last day of monitoring were found to be in the range of ±10 mV to ±30 mV, which is associated with incipient stability in the emulsion [42]. According to the statistical analysis, no significant differences were observed between samples on the tested days. This incipient stability made it necessary to monitor the lycopene concentration over storage time.

The destabilization of the Pickering emulsion can lead to degradation of the lycopene in the oil phase because it has a highly unsaturated structure that is prone to chemical degradation when exposed to light, elevated temperatures, oxygen, free radicals, and transition metals [40]. For this reason, we continued to monitor the lycopene concentration during the 15 days of storage. A first indication of changes in lycopene concentration can be given by monitoring and verifying the color of the product. For this reason, the color of the dressing was measured as shown in Table 5. Color results indicate that the three salad dressings exhibited slight color variations, but were predominantly characterized by white brilliance and colorations that leaned toward red and orange hues. This was attributed to the presence of lycopene in the sunflower oil utilized for the preparation of the dressing [43]. In addition, the color of the samples during the follow-up days changed with respect to the sample on day one. Nevertheless, the lack of the perception of color changes by the observer does not constitute a definitive conclusion regarding the occurrence of alterations in lycopene concentration.

**Figure 8:**
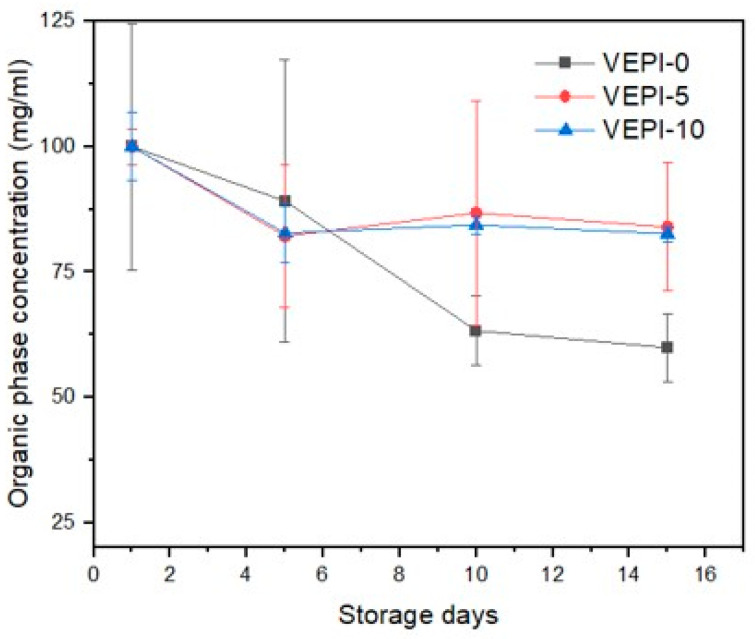
Variation in lycopene content in French-salad dressing during storage.

In order to ascertain whether a change has occurred in the concentration of lycopene, it is necessary to perform quantification during the storage period. Figure 8 shows the results of the determination of lycopene concentration. All salad dressings showed a decrease in lycopene concentration during the storage time, but no statistically significant changes were observed among them. The decrease in lycopene concentration was due to emulsion destabilization. The emulsion is used as a mechanism for lycopene protection, and it has been reported that unlike biopolymers and surfactants, which are also used in emulsion formulation, colloidal particles are strongly adsorbed on the surfaces of droplets and form a relatively thick rigid layer, which can improve the physical stability of emulsions, but also improve the chemical stability of encapsulated active ingredients [44]. Qi et al. (2020) [35] reported similar results for β-carotene encapsulated in a Pickering emulsion with wheat gluten nanoparticles; emulsion destabilization reduced the concentration of β-carotene by up to 80% when the emulsion was stored at 60 °C [35].

In order to assess the potential marketability of the product, a sensory analysis was conducted by an untrained panel of consumers. As can be seen in Table 6, the addition of vinegar resulted in an increase in the overall acceptability of the dressing; the sample with higher vinegar content, VEPI-10, showed greater overall acceptability. In addition, there were differences in the perception of color, odor, viscosity, taste, and residual flavor. The sensory characteristics observed for the color and texture of the French-salad dressing were related to the results obtained in the determination of the color and viscosity of the samples. In addition, the viscosity values for all samples were within the range reported as acceptable for dressing, which was associated with the positive assessment of viscosity by the panelists.

The results of this study indicate that this novel French-style salad dressing, offers an alternative source of lycopene, which may be beneficial in certain contexts. Nevertheless, further investigation is required to ascertain the impact of additional components, such as vinegar or salt, on the stability of Pickering emulsions as well as a deep analysis of surface phenomena. Furthermore, examining the oxidative stability may provide a better understanding of the shelf life of French-salad dressings.

## 3. Materials and Methods

The cellulose nanofibers (CNFs) used in this research were isolated from banana rachis following the KOH-5 protocol proposed by Zuluaga et al. (2009) [45]. The sunflower oil (SFO) enriched with lycopene was produced as reported by Hoyos et al. (2022) [15]. Commercial SFO, fruit vinegar, commercial white granular sugar, and table salt were purchased from a local supermarket in Medellín, Antioquia (Colombia). The organic volatile solvents including hexane, ethanol, and acetone were analytical grade and acquired from Merck (Darmstadt, Germany); an analytical standard of lycopene from Sigma-Aldrich (St. Louis, MO, USA) was used for the determination of lycopene content.

### 3.1. Preparation of Pickering Emulsions and Salad Dressings

Pickering emulsions and French-style salad dressings were produced using two sequential mechanical treatments: ultraturrax (UT) (IKA, Staufen, Germany), impeller model S50N-G45M, followed by a high-pressure homogenizer (HPH) (Panda2K, N1001L, NiroSoavi, Parma, Italy).

To process the Pickering emulsions and French-style salad dressing, the lycopene enriched sunflower oil, CNFs, and water were mixed in UT at 10,000 rpm for 2 min, subsequently, the samples were homogenized in HPH using both valves R and PS at a total pressure of 300 bar. After manufacturing, the samples were stored in a refrigerator at 3 °C under reduced lighting conditions to be characterized.

First, the influence of the CNFs on Pickering emulsion (samples named such as EPI) was analyzed. For this reason, three concentrations were evaluated and corresponded to CNFs 0.1 (EPI-0.1), 0.25 (EPI-0.25), and 0.5 wt.% (EPI-0.5) using a ratio of oil in water (O/W) of 1:9 [36].

Once the CNF concentration was established, three different formulations of French-style salad dressings (named VEPI) with lycopene-enriched SFO were evaluated. Table 7 summarizes the ingredient proportions used in the formulations of the Pickering emulsions and French-style dressings.

**Figure 9:**
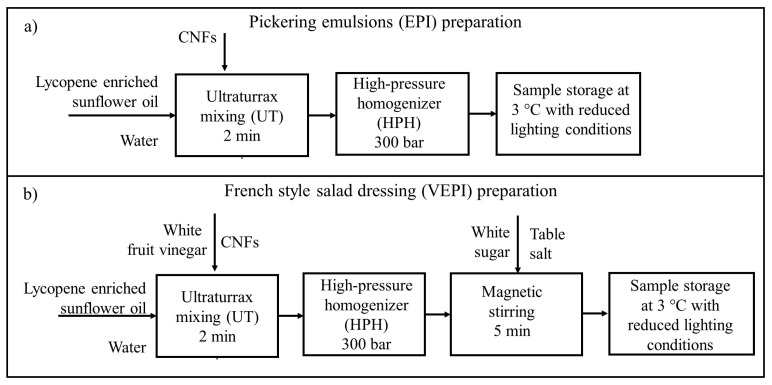
Elaboration of (**a**) Pickering emulsion (EPI) and (**b**) French-style salad dressing.

For the preparation of this dressing, a mixture of vinegar and water was thoroughly mixed with a CNF suspension and homogenized with lycopene-enriched SFO, as described for the obtention of Pickering emulsions. Finally, salt and sugar were added. The salad dressings obtained were stored in a refrigerator at 3 °C under reduced lighting conditions to be characterized. Figure 9 describes the steps followed to produce the Pickering emulsions and French-style salad dressings.

### 3.2. Characterization of Pickering Emulsions and Salad Dressings

#### 3.2.1. Physical Stability

To identify the appropriate CNF concentration to formulate a stable Pickering emulsion, the response variable defined was the physical stability of the emulsion, evaluated by the method described by Serpa et al. (2020) [33], with some modifications. Forty-five mL conical tubes were filled with each of the samples prepared and stored at 25 °C for 15 d for photographic monitoring to determine the time required for phase separation (Serpa et al., 2020) [33]. The same procedure was followed to study the stability of the salad dressing. Photographs were taken in a portable LED studio with a black background (n PULUZ^®,^ Dongguan City, China) using a 5 MP, f/2.0 PDAF camera (Xiaomi, Beijing, China).

For salad dressing, the stability index (SI) was calculated using Equation (1), where Vstable corresponds to the volume of the stable layer of the suspension, whereas Vtotal is the total volume [46].

(1) SI%=Vstable/Vtotal

#### 3.2.2. Microstructure

Oil and water phases in the Pickering emulsions and salad dressing were investigated by fluorescence microscopy (Zeiss Axio Observer, Zeiss, Wetzlar, Germany) coupled to a light source HXP 120 at a magnification of 40×. For this analysis, two chromophores were used, Nile red for lipophilic stains with extinction and emission wavelengths at 488 and 539-641 nm, respectively, and Calcofluor white, which binds to nanocellulose, in this case, excitation and emission wavelengths of 488 nm and 506 nm, respectively [47]. For the analysis, 1 mL of sample was stained with 8 μL Nile red diluted in ethanol (1 mg/mL), followed by adding 6 μL of Calcofluor white; 4 μL of the prepared samples was used for the study [20]. For each sample, three types of images were taken: one in brightfield microscopy to observe the microstructure of the emulsion and two fluorescence images to observe the oil phase and nanocellulose of the emulsion. The obtained images were merged into one using FIJI 2.9.0 [48] to have a clearer observation of the microstructure of the Pickering emulsion.

#### 3.2.3. pH Evaluation

The pH of the salad dressing was assessed on days 1, 5, 10, and 15 using a previously calibrated SCHOTT brand pH meter. Triplicate measurements were made per sample and the mean and standard deviation were reported.

#### 3.2.4. Rheological Analysis

The emulsion that showed the greatest stability over time was studied for flow and viscoelastic behavior on days 1, 5, 10 and 15 using an HR-2 rheometer (T.A. Instruments, New Castle, Delaware, USA) with a plate-plate geometry at 4 °C [49] and a gap of 1 mm.

Tests were performed in triplicate; data acquisition and processing were performed with TRIOS software (TA Instruments Ltd., New Castle, Delaware, USA). Shear stress was recorded at increasing shear rates from 0.1 to 100 s^−1^. The curves obtained were fitted to the Ostwald-de Waele model. To determine the viscoelastic properties, a frequency sweep was performed from 1 to 10 rad/s, recording the loss (G″) and storage (G′) moduli [50].

The French-style salad dressing was evaluated using the same criteria and conditions as for the emulsions.

#### 3.2.5. Z Potential and Particle Size

The surface charge of the stabilized emulsions was measured since the day of preparation until day 15. Measurements were performed after five days of storage using dynamic light scattering (DLS). A Zetasizer Pro (Malvern Instruments Ltd., Worcestershire, UK) was used for the Z-potential measurement using a Z potential cell of the same provider [36]. Refraction indices used during measurement were 1.46 and 1.33 for sunflower oil and water, respectively [30].

The French-style salad dressing was tested under the same criteria and conditions as the emulsions.

#### 3.2.6. Lycopene Content

Quantification of lycopene content on the Pickering emulsions and salad dressing samples was performed immediately after its preparation (day 1) and after 5, 10, and 15 d of storage. The quantification was performed using UV–Vis spectrophotometry. The extraction was performed by mixing 2 mL of the emulsion with 2 mL of an extraction solution (hexane:acetone:ethanol, 2:1:1) for 1 min at 3000 rpm. The mixture was centrifuged for 10 min at 6000 rpm to separate the organic phase that was completed to 10 mL in a volumetric flask. A total of 1 mL of the organic phase was taken with the aid of a micropipette and made up to 2 mL with extraction solution and mixed in vortex at 3000 rpm for 10 s. Finally, the absorbance was measured at 473 nm in a UV–Vis spectrophotometer (Evolution 600, Thermo Scientific, Waltman, MA, USA). The same process was performed on emulsions with sunflower oil without lycopene to obtain a spectrophotometric blank. The calibration curve (R^2^ = 0.987) was plotted for a standard solution of lycopene [51,52].

#### 3.2.7. Color Difference

The color difference of the Pickering emulsions and salad dressings samples on days 1, 5, 10, and 15 after preparation was determined using Equation (2). The coordinates of the samples on day 1 were taken as the control (L_o_*, a_o_*, and b_o_*). Color was measured using CIEL coordinates* a* b*. These values are based on the opposite color model, where (L*) corresponds to brightness, (a*) is the intensity of the green color (positive values) to red (negative values), and (b*) is the intensity of the yellow color (positive values) to red (negative values) [53].

(2) $$∆E=\sqrt{(L^{*}-{L_{O}}^{*}{)}^{2}+({a^{*}-{a_{0}}^{*})}^{2}+({b^{*}-{b_{0}}^{*})}^{2}}$$

#### 3.2.8. Sensory Analysis

To evaluate the sensory properties of salad dressings, an acceptance sensory analysis was performed with a 9-point hedonic scale, being 1—extremely dislike and 9—extremely like [54,55,56]. Thirty untrained panelists with ages ranging from 19 to 58 were recruited from the Universidad Pontificia Bolivariana at Medellin, Colombia. The evaluated attributes were color, flavor, texture, aroma, after taste, and overall acceptability. Samples were given to the panelist in plastic cups (20 mL) coded with a three digit code. The panelists used water and unsalted cookies to clean their palates between samples [57]. Given that the evaluated salad dressings included a nanosized component (CNFs), an explanation of its implications in terms of normative and safety was given to each panelist before the assay, and to continue, each one had to sign an informed consent. This consent was made based on the policies of the Research Ethics Policy, Bioethics, and Scientific Integrity in Colombia [58] (C-8).

Participants were properly informed about the purpose, procedures, and potential risks of the study. They were assured that their participation was voluntary and that they could withdraw from the study at any time without any consequences. All data collected were anonymized to ensure the confidentiality and privacy of the participants. In light of this method, the Institution’s Ethical Committee did not request any new requirements.

### 3.3. Statistical Analysis

Experimental tests were performed in triplicate. Results are given as the mean of the three measurements and the standard deviation. Statistical analysis was performed using one-way analysis of variance (ANOVA) using Statgraphics Centurion 18 (Statgraphics Technologies Inc., The Plains, VA, USA). Significance was determined at 95% of confidence. A *p*-value < 0.05 was considered significant.

The Student’s *t*-test was performed on the Z potential results for French-style salad dressing, as presented in Table 4.

## 4. Conclusions

In this study, a novel French-style salad dressing was developed using a Pickering emulsion based on lycopene extracted from guava and nanocellulose isolated from banana residues. In the initial phase of the study, optical and rheological analysis revealed that cellulose nanofibers play a pivotal role in maintaining the stability of the Pickering emulsion. However, the stability of the dressing is affected during storage time, particularly due to the interaction with other components such as vinegar and salt, thereby destabilizing the network of cellulose nanofibers deposited at the oil–water interface. The destabilization of the emulsion resulted in color changes that were not perceptible to the human eye but were associated with a reduction in lycopene concentration, which decreased to 60% of the initial value. Ultimately, the sensory analysis indicated that the product was well-received and aligned with the observed changes in color and viscosity. These outcomes provide a foundation for further investigation into the potential applications of this Pickering emulsion in food products, particularly in light of the observed interactions with other components that can affect the networking of cellulose nanofibers.

As mentioned, the addition of vinegar to salad dressings showed a decrease in lycopene concentration during storage but increased the overall acceptability of the dressing; the sample with the higher vinegar content, VEPI-10, showed greater overall acceptability.

## Figures and Tables

**Table 1:** Parameters of the Ostwald-de-Waele model for the Pickering emulsion EPI0.5 sample.

Storage Time	K (Pa∙s^n^)	n	R^2^
Day 1	0.472 ± 0.001 ^a^	0.738 ± 0.011 ^a^	0.998
Day 15	0.575 ± 0.040 ^b^	0.483 ± 0.019 ^b^	0.986

^a, b^ Values with different superscript letters at day 1 and day 15 were significantly different (*p* < 0.05).

**Table 2:** The ζ potential, particle size lycopene content, and color change of the EPI0.5 emulsion.

Storage Time	Property	Units	Value
Day 1	ζ potential	mV	−36.380 ± 3.352 ^a^
	D [3,2]	μm	19.125 ± 1.096 ^a^
	Lycopene content	mg/L	3.933 ± 1.432 ^a^
Day 15	ζ potential	mV	−29.125 ± 0.488 ^b^
	D [3,2]	μm	20.800 ± 3.252 ^a^
	Lycopene content	mg/L	2.374 ± 0.720 ^a^
	Total color change (∆E)		2.617 ± 0.314 ^a^

^a, b^ Values with different superscript letters at day 1 and day 15 were significantly different (*p* < 0.05).

**Table 3:** Parameters of Ostwald-de-Waele model for the French-salad dressing sample.

Parameter	VEPI-O	VEPI-5	VEPI-10
K (Pa∙s^n^)	1.022 ± 0.024 ^a^	1.573 ± 0.073 ^b^	1.230 ± 0.025 ^c^
n	0.747 ± 0.011 ^a^	0.770 ± 0.004 ^b^	0.695 ± 0.009 ^c^
R^2^	0.999	1.000	0.996

Values with different superscript letters in the same row were significantly different (*p* < 0.05).

**Table 4:** Evaluation of the ζ potential results for French-style salad dressing during storage.

Sample	Day 1 (mV)	Day 5 (mV)	Day 10 (mV)	Day 15 (mV)
ζ Potential (mV)				
VEPI-0	−28.68 ± 8.04	−17.6 ± 9.68	−19.41 ± 9.43	−26.4 ± 7.80
VEPI-5	−18.9 ± 5.82	−19.48 ± 7.49	−18.25 ± 9.71	−25.02 ± 9.26
VEPI-10	−36.57 ± 8.16	−18.04 ± 9.18	−10.91 ± 8.91	−25.1 ± 7.18

**Table 5:** Color change of the French-style salad dressings.

Samples	Days	L*	a*	b*	∆E
**VEPI-0**	**1**	87.766 ± 1.843 ^a^	9.518 ± 0.539 ^a^	11.866 ± 0.452 ^a^	n.a
	**5**	88.408 ± 0.760 ^a^	8.336 ± 0.344 ^b^	11.522 ± 0.350 ^a^	1.324 ± 0.526 ^a^
	**10**	88.668 ± 0.757 ^a^	6.978 ± 0.195 ^c^	10.912 ± 0.164 ^b^	2.617 ± 0.314 ^b^
	**15**	89.135 ± 0.231 ^a^	7.215 ± 0.044 ^d^	10.510 ± 0.110 ^a^	2.495 ± 0.103 ^b^
**VEPI-5**	**1**	91.744 ± 0.329 ^a^	8.440 ± 0.237 ^a^	10.414 ± 0.280 ^a^	n.a
	**5**	91.992 ± 0.489 ^a^	7.652 ± 0.154 ^b^	9.916 ± 0.140 ^b^	1.090 ± 0.236 ^a^
	**10**	92.350 ± 0.123 ^a^	6.162 ± 0.385 ^c^	9.832 ± 0.434 ^b^	2.518 ± 0.517 ^b^
	**15**	92.594 ± 0.192 ^a^	5.286 ± 0.052 ^d^	9.600 ± 0.084 ^b^	3.407 ± 0.263 ^c^
**VEPI-10**	**1**	91.118 ± 0.107 ^a^	8.792 ± 0.069 ^a^	10.282 ± 0.066 ^a^	n.a
	**5**	91.700 ± 0.519 ^b^	8.478 ± 0.233 ^a^	9.932 ± 0.253 ^b^	0.903 ± 0.363 ^a^
	**10**	93.264 ± 0.249 ^c^	8.670 ± 0.166 ^a^	10.764 ± 0.098 ^c^	2.205 ± 0.197 ^b^
	**15**	93.338 ± 0.338 ^c^	8.460 ± 0.107 ^a^	11.388 ± 0.243 ^d^	2.531 ± 0.090 ^b^

Values with different superscript letters in the same column were significantly different (*p* < 0.05).

**Table 6:** Sensorial evaluation of French-style salad dressing.

Sample	Color	Odor	Viscosity	Flavor	Residual Flavor	Overall Level of Acceptance
**VEPI-0**	6.7 ± 1.4	5.9 ± 1.4	6.7 ± 1.7	5.6 ± 2.0	5.8 ± 1.7	5.9 ± 1.7
**VEPI-5**	6.9 ± 1.2	6.6 ± 1.5	7.2 ± 1.4	6.1 ± 1.7	6.0 ± 1.4	6.1 ± 1.8
**VEPI-10**	6.9 ± 1.2	6.7 ± 1.4	7.3 ± 1.3	6.2 ± 1.5	6.3 ± 1.5	6.7 ± 1.2

**Table 7:** Ingredient proportions used in the formulations of the Pickering emulsions and French-style salad dressing.

Sample	EPI-0.1	EPI-0.25	EPI-0.5	VEPI-0	VEPI-5	VEPI-10
**Vinegar (g)**	0	0	0	0	5	10
**Water (g)**	86.3	80.8	71.5	62	57	52
**SFO enriched with lycopene (g)**	10	10	10	10	10	10
**CNFs 2 wt.% (g)**	3.7	9.2	18.5	25	25	25
**Salt (g)**	--	--	--	1	1	1
**Sugar (g)**	--	--	--	2	2	2

## Data Availability

The data reported in this study are included in the journal, with the exception of the sensory test, which is subject to ethical restrictions.

## Supplementary Materials

The following supporting information can be downloaded at: https://www.mdpi.com/article/10.3390/molecules29215118/s1, Figure S1: Cellulose nanofibers (CNFs) isolated from banana rachis utilized in this work.
