# Supplementary material for: A Novel French-Style Salad Dressing Based on Pickering Emulsion of Oil-Water Lycopene from Guava and Cellulose Nanofibers
Source: Molecules. 2024 Oct 30;29(21):5118. doi: 10.3390/molecules29215118 (PMC11547640; doi:10.3390/molecules29215118)
Supplement: Supplementary file 1 [file molecules-29-05118-s001.zip › molecules-3257493-supplementary.pdf]

A Novel French-Style Salad Dressing Based on Pickering Emulsion of Oil-Water Lycopene from Guava and Cellulose Nanofibers

Catalina Gómez-Hoyos [1,*], Angélica Serpa-Guerra [2], Shaydier Argel.Pérez [1],

Jorge Andrés Velásquez.Cock [1], Lina Vélez-Acosta [2],

Piedad Gañán-Rojo [3,*] and Robin Zuluaga-Gallego [2]

[1]Programa de Ingeniería en Nanotecnología, Universidad Pontificia Bolivariana, Medellín Colombia

[2]Facultad de Ingeniería Agroindustrial, Universidad Pontificia Bolivariana, Medellín Colombia.

[3]Facultad de Ingeniería Química, Universidad Pontificia Bolivariana, Medellín Colombia.

*Correspondence: catalina.gomezh@upb.edu.co (C.G.-H.); piedad.ganan@upb.edu.co (P.G.-R.);

Supplementary information

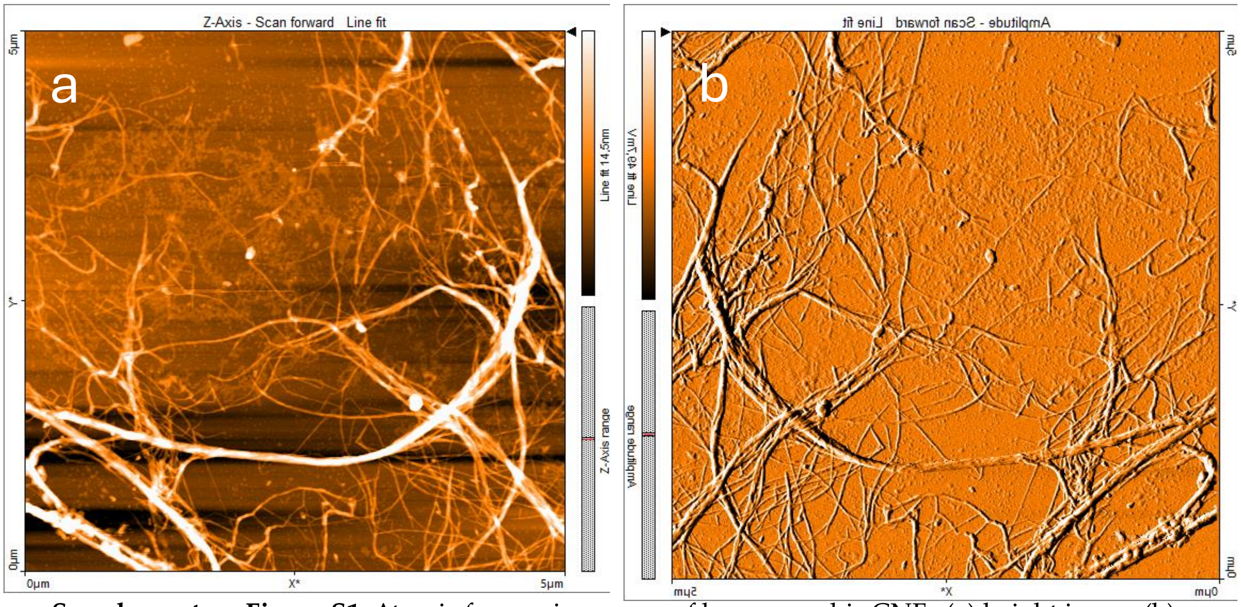

Supplementary Figure S1. Atomic force microscopy of banana rachis CNFs (a) height image (b) amplitude image
